# Overexpression of Optic Atrophy Type 1 Protects Retinal Ganglion Cells and Upregulates Parkin Expression in Experimental Glaucoma

**DOI:** 10.3389/fnmol.2018.00350

**Published:** 2018-09-28

**Authors:** Xinxin Hu, Yi Dai, Rong Zhang, Kunte Shang, Xinghuai Sun

**Affiliations:** ^1^Department of Ophthalmology and Visual Science, Eye and ENT Hospital, Shanghai Medical College, Fudan University, Shanghai, China; ^2^NHC Key Laboratory of Myopia, Fudan University, Shanghai, China; ^3^Key Laboratory of Myopia, Chinese Academy of Medical Sciences, Shanghai, China; ^4^Shanghai Key Laboratory of Visual Impairment and Restoration, Fudan University, Shanghai, China; ^5^State Key Laboratory of Medical Neurobiology, Institutes of Brain Science, Collaborative Innovation Center for Brain Science, Fudan University, Shanghai, China

**Keywords:** OPA1, parkin, retinal ganglion cells, mitochondria, glaucoma

## Abstract

Glaucoma is a neurodegenerative disease that features progressive loss of retinal ganglion cells (RGCs). Increasing evidences have revealed that impaired mitochondrial dynamics occurs early in neurodegenerative diseases. Optic Atrophy Type 1 (OPA1), a mitochondrial fusion protein, has recently been suggested to be a mitophagic factor. Our previous studies found that glaucomatous retinal damage may be ameliorated by an increase in mitochondrial OPA1. In this study, we explored the mechanism involved in OPA1 mediated neuroprotection and its relationship with parkin dependent mitophagy in experimental glaucoma models. Our data showed that overexpression of OPA1 by viral vectors protected against RGC loss, attenuated Bax expression, and improved mitochondrial health and mitochondrial surface area. Parkin expression and the number of mitophagosomes were upregulated in OPA1 overexpressed RGCs under glutamate excitotoxicity. While knockdown of OPA1 by siRNA decreased protein expression of parkin in RGCs under glutamate excitotoxicity. Two weeks after intraocular pressure (IOP) elevation, the LC3-II/I ratio and the LAMP1 expression were increased in OPA1 overexpressed optic nerve. These findings suggest that OPA1 overexpression may protect RGCs by ways of enhancing mitochondria fusion and parkin mediated mitophagy. Interventions to promote mitochondrial fusion and mitophagy may provide a useful strategy to battle against glaucomatous RGC loss.

## Introduction

Glaucoma is a complex, multifactorial neurodegenerative disease that features progressive loss of retinal ganglion cells (RGCs) (Gupta and Yucel, 2007). Evidences have shown that impaired mitochondrial dynamics may play an important role in glaucomatous RGC loss (Ju et al., 2008; Dai et al., 2011). Optic Atrophy Type 1 (OPA1), a dynamin-related GTPase located in the inner mitochondrial membrane, is required for mitochondria fusion and cristae morphology (Itoh et al., 2013). Mutations in nuclear gene OPA1 is the culprit of autosomal dominant optic atrophy, a major form of inherited optic neuropathy, which features a progressive loss of RGCs that leads to legal blindness. Previous studies indicated that OPA1 is present in the axons and soma of the RGCs, as well as in horizontal cells of rodent’s retinae (Ju et al., 2005; Ju et al., 2009). Down-regulation of OPA1 results in mitochondrial aggregation in purified RGCs (Kamei et al., 2005), while increased OPA1 expression may ameliorate glaucomatous retinal damage (Ju et al., 2010; Dai et al., 2011). Nevertheless, the mechanisms underlying how OPA1 can play a protective role to glaucomatous RGCs remain largely unknown.

It has been suggested that mitochondrial dynamics including mitochondrial fusion and fission play key roles in mitophagy-related mitochondrial quality control (Ni et al., 2015). Parkin, an E3 ubiquitin ligase (Truban et al., 2017), targets damaged mitochondria for degradation by autophagosomes (Youle and Narendra, 2011). Increasing evidences have revealed the significant linkage between parkin-dependent mitophagy and neurodegenerative diseases, including Alzheimer’s and Parkinson’s diseases. Recently, our study has demonstrated that parkin overexpression played a prominent protective role in RGCs, and partially restored the malfunction of mitophagy in experimental glaucoma (Hu et al., 2017; Dai et al., 2018). Overexpression of parkin induced upregulation of the OPA1 protein in RGCs with moderate mitochondrial dysfunction, while OPA1 expression was decreased when the mitochondria are irreversibly damaged by cumulative IOP elevation.

Hence, the present study was undertaken to address whether overexpression of OPA1 can protect RGCs in experimental glaucoma models, and the mechanism involved in OPA1 mediated mitochondrial fusion and parkin dependent mitophagy.

## Materials and Methods

### Animals

All procedures concerning animals are performed in compliance with the ARVO Statement for the Use of Animals in Ophthalmic and Vision Research. The study protocol was reviewed and approved by the Animal Ethics Committee of the Eye and ENT Hospital of Fudan University, China. Male Sprague-Dawley rats (240–300 g in weight) were raised in standard cages under constant 12-h light/dark cycles and supplied with water *ad libitum* and standard rodent diet.

### Cultured Retinal Ganglion Cell Culture and Treatment

Retinal ganglion cells were isolated and cultured as previously described (Hu et al., 2017). Briefly, retinas from 2- to 3-day-old Sprague-Dawley rats were dissociated in 5 mg/ml of papain solution (Worthington Biochemical, Lakewood, NJ, United States). The retinal suspensions were then sequentially incubated with a petri dish coated with rabbit anti-macrophage antibody (Millipore Corp., Billerica, MA, United States) and mouse anti-Thy1.1 antibody (Abcam, Cambridge, MA, United States). RGCs were seeded into appropriate plates coated with 0.01% poly-D-lysine (Sigma-Aldrich, St. Louis, MO, United States). Adenovirus, designed and packaged by (Sunbio, Shanghai, China) as previously described (Hu et al., 2017), were diluted in cell culture medium for 48 h to infect RGCs. The RGCs were then incubated with 100 μM glutamate (Sigma-Aldrich) to induce excitotoxicity model.

Small interfering RNA (siRNA) targeted against OPA1 was designed and packaged by Genomeditech Co., Ltd. (Shanghai, China). The sequences used were as follows: 5′- GACAUCUUUUCAGCAAUUC-3′. Transfection was performed with RNAi max (Thermo Fisher Scientific, Shanghai, China) according to the manufacturer’s instruction. Seventy-two hours after transfection, the RGCs were then incubated with 0 μM or 100 μM glutamate as described above.

### Quantitative PCR

Total RNA from RGCs (*n* = 3 groups) was extracted with Trizol (Invitrogen, Carlsbad, CA, United States) according to the manufacturer’s instructions. The target gene was amplified by qPCR (SYBR; Takara, Tokyo, Japan) with a program (95°C for 15 s, and 60°C for 30 s for 45 cycles). GAPDH was used as endogenous reference. The data were analyzed using the 2^-ΔΔ^*^C^*^t^ method.

### Measurement of Cytotoxicity

The amount of lactate dehydrogenase (LDH) released from cytoplasm of damaged RGCs was performed using the LDH Cytotoxicity Detection Kit (TaKaRa Biotechnology, Dalian, China) according to the manufacturer’s instructions. After each treatment, the supernatant media of RGCs is collected and incubated with the reconstituted substrate mix. Absorbance was measured at 490 nm using a microplate reader (Synergy H1, BIOtAK).

### Assessment of Cell Apoptosis

Apoptosis of RGCs was measured with Hoechst staining. After being fixed with 4% paraformaldehyde (PFA) in PBS for 20 min at room temperature, RGCs on the coverslips were permeabilized with 0.1% Triton X-100 for 20 min at room temperature. Cells were then incubated with Hoechst 33,342 (1 μg/ml, Life Technologies, Grand Island, NY, United States) for 10 min at room temperature. Images were acquired randomly using a confocal microscope (Leica SP8, Mannheim, Germany). The percentage of apoptotic cells with pyknotic nuclei was calculated for each group.

### Measurement of Mitochondrial Membrane Potential

Mitochondrial membrane potential was assessed using the JC-1 Assay Kit (Abcam). RGCs with different treatments were incubated with JC-1 (5 μg/ml) dye for 20 min at 37°C. JC-1 fluorescence intensities of red aggregates (hyperpolarization) and green fluorescence monomers (depolarization) were read with a fluorescent plate reader (Infinite M1000; Tecan, Mnnedorf, CH) as previously described (Hu et al., 2017). The experiments were performed in triplicate.

### Mitochondria Distribution

Mitochondria distribution: RGCs were loaded with 200 nM MitoTracker Red (Molecular Probes, M7512; Life Technologies) for 30 min at 37°C, and then were washed for three times. RGCs grown on cover slips were fixed with 4% PFA in PBS for 10 min at room temperature, and then were incubated in 0.2% Triton X-100 for 5 min. Fluorescence imaging were performed with a confocal microscope (Leica SP8).

### Experimental Glaucoma

Chronic experimental glaucomatous model was induced by translimbal laser photocoagulation of the trabecular meshwork as previously described (Dai et al., 2011). All rats were injected with a mixture of ketamine (80 mg/kg, Gutian Pharmaceutical Co., Ltd., Fujian, China) and xylazine (5 mg/kg, Sigma-Aldrich, St. Louis, MO, United States) for anesthesia. Rat eyes were delivered approximately 60–100 trabecular burns distributed around the limbus by argon laser (532 nm wavelength, 300 mW power, 0.5-s duration, 50-mm diameter spot size, Coherent ULTIMA2000, United States) after topical application of 0.4% oxybuprocaine hydrochloride (Santen) drop.

Elevated intraocular pressure (IOP) was measured in each eye under anesthesia once before laser treatment, 1, 3, and 7 days after each treatment using a hand-held tonometer (TonoLab; Tiolat Oy, Helsinki, Finland). If hemorrhage or anterior chamber inflammation was observed by slit lamp ophthalmoscopy, rat was excluded from further experiment. The laser treatment was repeated after 1 week for all rats except those killed at 3 and 7 days.

### Plasmid, Recombinant AAV2 Constructs and Injection of AAV2 Vectors

AAV2-GFP-OPA1 and AAV2-GFP-null were designed and packaged by (Sunbio, Shanghai, China) as previously described (Dai et al., 2018). The titers of AAV2-GFP-OPA1 and AAV2-GFP-null were 1.74 × 10^12^ and 5.13 × 10^12^ vector genomes (v.g.)/ml, respectively.

Five microliters of AAV2-GFP-OPA1 or AAV2-GFP-null containing 5 × 10^9^ units of AAV2 virus were injected into the center of the vitreous via a 33-gauge Hamilton syringe (Hamilton Company, Reno, NV, United States). In order to minimize loss of virus through the injection tract, AAV2 were delivered slowly over 1 min and the syringe was kept in position for another 1 min. Rats were excluded with punctured lens or damaged retina.

### Retrograde Labeling of RGCs

Four days before the first laser treatment, 2 μl of 5% of FluoroGold (FluoroGold; Sigma-Aldrich, St. Louis, MO, United States) was microinjected into the superior colliculus on each side of anesthetized rats, as described previously (Gao et al., 2017). Eyes obtained from the rats 2 weeks after IOP elevation were fixed in 4% paraformaldehyde in PBS and prepared as flat-mount. Images of whole retinas were captured with a confocal microscope (Leica SP8). RGCs were quantified and averaged in the central retina (1–2 mm from the optic disk), in the middle retina (2–3 mm from the optic disk) and in the peripheral retina (3–4 mm from the optic disk).

### Transmission Electron Microscopy

The optic nerve heads of three rats from each group were separated on ice and immediately fixed in 2.5% glutaraldehyde (Ted Pella, Redding, CA, United States) in 0.1 M phosphate buffer at 4°C. After rinsing in 0.1 M phosphate buffer for three times, tissues were post-fixed in 1% osmic acid at 4°C for 2 h, washed for three times, dehydrated in ascending grades of ethyl alcohol, and embedded in epoxy resin. Then sections on unmyelinated optic nerve were sliced and then viewed using a transmission electron microscope.

### Western Blot Analysis

Retinas (*n* = 3 per group) were lysed in RIPA buffer (Beyotime, China) and ultrasonically smashed on ice to get homogenized solutions. RGCs (*n* = 3 per group) were mixed with RIPA buffer. Equal amounts of protein were separated by sodium dodecyl sulfate-polyacrylamide gel electrophoresis (SDS-PAGE) and the electrotransferred onto polyvinylidene difluoride membranes (Millipore, Billerica, MA, United States). After blocking with 5% non-fat dry milk at room temperature for 1 h, the membranes were incubated with polyclonal rabbit anti-OPA1 (1:1000; Abcam), polyclonal rabbit anti-GFAP antibody (1:10000; Abcam), polyclonal rabbit anti-Parkin (1:1000; Abcam), polyclonal rabbit anti-Bcl-2 (1:500; Abcam), monoclonal rabbit anti-Bax (1:1,000; Abcam), monoclonal rabbit anti-LC3 (1:2000; Abcam), polyclonal rabbit anti-LAMP1 (1:1000; Abcam) and polyclonal rabbit anti-GAPDH (1:2000; Yesen, China) in primary antibody dilution (Beyotime, China) overnight at 4°C. After the membranes were washed several times, secondary antibody peroxidase-conjugated goat anti-rabbit IgG (1:5000; Jackson) was applied. Proteins were visualized by a Kodak Image Station 4000MM PRO (Carestream, Rochester, NY, United States) using chemiluminescence detection (SuperSignal West Femto Substrate Trial Kit, Thermo Fisher). Band intensity was analyzed with Image J (National Institutes of Health).

### Immunohistochemistry Analysis

Immunohistochemical staining of 7-um frozen sections of full-thickness retina was prepared. Three sections per frozen block from each group (*n* = 3 retinas/group) were used for immunohistochemical analysis. The RGCs (*n* = 3 per group) on the coverslips were fixed with 4% PFA in PBS for 20 min, rinsed with PBS, and then were used for immunohistochemical analysis. The samples were permeabilized with 0.1% Triton X-100 in PBS for 20 min at room temperature, sequentially, blocked with 5% bovine serum albumin (BSA) for 1 h at room temperature, incubated by 16 h at 4°C with the following primary antibodies: monoclonal mouse anti-GFAP antibody (1:200; Abcam), polyclonal rabbit anti-OPA1 (1:200; Abcam), and monoclonal rabbit anti-LC3 (1:1000; Abcam) for 16 h at 4°C. After several washes, the samples were incubated with Alexa Fluor 488-conjugated goat IgG secondary antibody (1:200; Life Technologies) and Hoechst 33342 (1 μg/mL; Life Technologies). Fluorescence imaging and analyses were performed by a confocal microscopy (Leica SP8).

### Statistical Analysis

All data are expressed as mean ± SD. All experiments were repeated at least three times. Data analysis was performed using one-way analysis of variance and the Bonferroni *t*-test. *P*-value < 0.05 was considered statistically significant.

## Results

### Effects of OPA1 Overexpression on Cultured RGCs Under Glutamate Excitotoxicity

**FIGURE 1:**
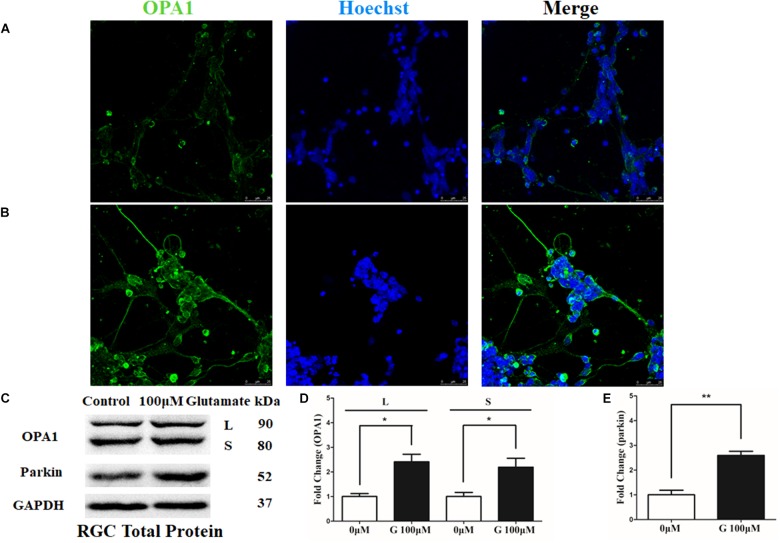
OPA1 expression in cultured RGCs under glutamate excitotoxicity. Compared with the control group **(A)**, increased OPA1 expression was observed in the cell bodies and axons of the RGCs after incubation with 100 μM glutamate **(B)**. Expression of OPA1 and parkin detected by Western blot in the RGCs control and 100 μM glutamate groups **(C–E)**. Compared with the control group, increased expression of the isoforms L and S of the OPA1 protein **(C,D)** and parkin protein were observed. *n* = 3, data are expressed as mean ± SD, ^∗^*p* < 0.05, ^∗∗^*P* < 0.01 compared with the control group. Scale bar = 25 μm **(A,B)**.

OPA1 protein was mainly expressed in the cell bodies and axons of cultured RGCs. In the excitotoxicity model of 100 μM glutamate treatment, OPA1 immunoreactivity was increased in the cell bodies and axons of the RGCs than that in the control RGCs (**Figures 1A,B**). Western blot results indicated that the protein levels of isoforms L (*P* < 0.05) and S (*P* < 0.05) of OPA1 were higher than those in control (**Figures 1C,D**). While the increased protein level of parkin was also observed in the excitotoxicity model of RGCs (**Figures 1C,E**).

**FIGURE 2:**
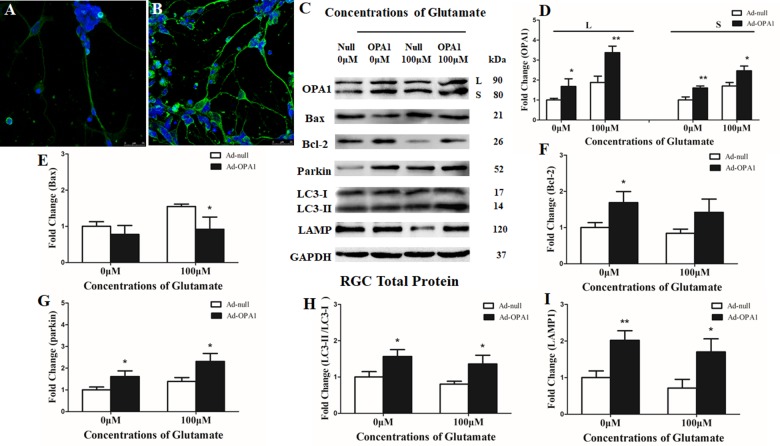
Effects of OPA1 overexpression on RGCs under glutamate excitotoxicity. Immunofluorescence of OPA1 expression in RGCs transfected with Ad null and Ad-OPA1 **(A,B)**. Compared with the Ad null transfected RGCs **(A)**, immunoreactivity of OPA1 were greater in the RGCs transfected with Ad-OPA1 **(B)**. Western blot analysis of the OPA1, Bax, Bcl-2, parkin, LC3-I and -II and LAMP1 proteins in OPA1-transfected RGCs **(C–I)**. Compared with Ad null transfected, expression of isoform L and S of the OPA1 protein in Ad-OPA1-transfected RGCs were increased in the 0 and 100 μM glutamate groups **(C,D)**, expression of Bax protein was decreased in 100 μM glutamate group **(C,E)**, while expression of Bcl-2 protein was increased in neurobasal medium group **(C,F)**, parkin expression was significantly increased in the 0 and 100 μM glutamate groups **(C,G)**, LC3-II/I ratio **(C,H)** and the expression of LAMP1 protein **(C,I)** were increased in the 0 and 100 μM groups. *n* = 3, data are expressed as mean ± SD, ^∗^*P* < 0.05, ^∗∗^*P* < 0.01 compared with Ad null transfected groups. Scale bar = 25 μm **(A,B)**.

Immunoblotting after transfection for 2 days was used to detect the efficiency of the OPA1 overexpression in RGCs. Upregulated expression of OPA1 protein (**Figures 2C,D**) and upregulated immunoreactivity of OPA1 (**Figures 2A,B**) were observed in the RGCs transfected with Ad-OPA1 (*P* < 0.05). In comparison with Ad null transfected RGCs in neurobasal medium, the protein levels of Bcl-2 were increased Ad-OPA1-transfected RGCs (*P* < 0.05, **Figures 2C,F**). No significant differences were observed for Bax protein level (*P* > 0.05, **Figures 2C,E**), JC-1 ratio and LDH activity between Ad-OPA1 and Ad null transfected RGCs without glutamate treatment.

**FIGURE 3:**
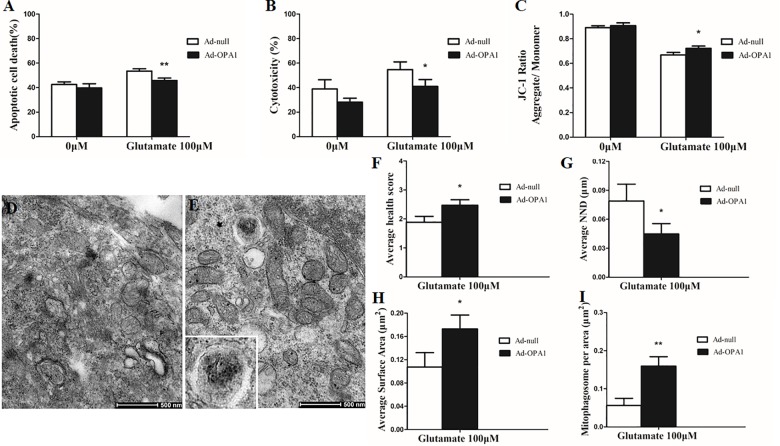
Ultrastructural analysis of OPA1 overexpression on RGCs under glutamate excitotoxicity. Compared with the Ad null transfected RGCs, the Ad-OPA1-transfected RGCs showed less apoptotic cell death **(A)**, a lower level of cytotoxicity **(B)** and a higher level of mitochondrial membrane potential **(C)** under glutamate excitotoxicity. Ultrastructural analysis of mitochondrial morphology and mitophagy in RGCs **(D–I)**. Compared with the Ad null transfected RGCs **(D)**, higher mitochondrial health scores **(E,F)**, closer nearest neighbor distance (NND) **(E,G)**, larger mitochondrial surface areas **(E,H)** and more number of mitophagosomes **(E,I)** were observed in the Ad-OPA1-transfected under glutamate excitotoxicity. *n* = 3, data are expressed as mean ± SD, ^∗^*P* < 0.05, ^∗∗^*P* < 0.01 compared with Ad null transfected groups. Scale bar = 500 nm **(D,E)**.

In the excitotoxicity model of 100 μM glutamate treatment to RGCs, it induced 16.18% of apoptotic cell. Compared with the Ad null transfected RGCs, Ad-OPA1-transfected RGCs had significantly decreased apoptotic cell by 18.1% under glutamate excitotoxicity (*P* < 0.01, **Figure 3A**). Ad-OPA1-transfected RGCs showed lower level of cytotoxicity than Ad null transfected RGCs by LDH measurements (*P* < 0.05, **Figure 3B**). Ad null transfected RGCs had a JC-1 ratio of 0.67 ± 0.02, whereas the JC-1 ratio of Ad-OPA1-transfected RGCs was 0.72 ± 0.02, which implied that overexpression of OPA1 increased the level of mitochondrial membrane potential under glutamate excitotoxicity (*P* < 0.05, **Figure 3C**). In comparison with Ad null transfected RGCs, western blot results indicated that protein level of Bax was decreased significantly in Ad-OPA1-transfected RGCs (*P* < 0.05, **Figures 2C,E**). However, no significant change was observed for the expression of Bcl-2 (*P* < 0.05, **Figures 2C,F**).

### Impacts of OPA1 Overexpression on Mitochondrial Morphology and Mitophagy in RGCs Under Glutamate Excitotoxicity

**FIGURE 4:**
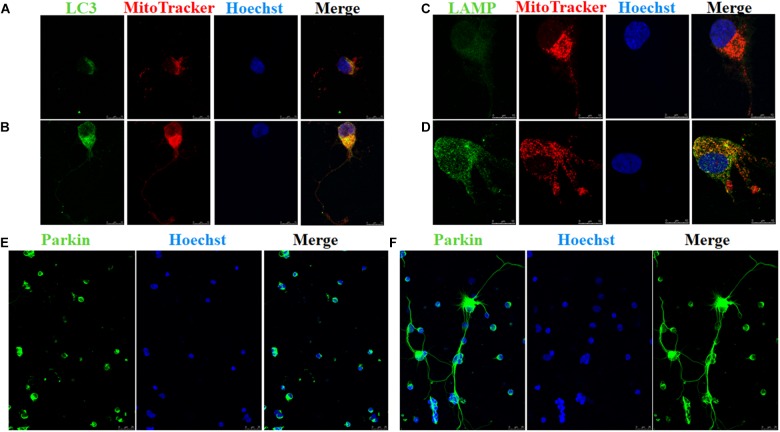
Immunofluorescence of RGCs after overexpression of OPA1 under glutamate excitotoxicity. RGCs co-stained with MitoTracker Red and LC3 or LAMP1 **(A–D)**. Glutamate treatment induced small spherical mitochondria and low immunoreactivity of LC3 and LAMP1 **(A,C)**. Overexpression of OPA1 induced more number of large mitochondria, reduced glutamate-induced mitochondrial fragmentation **(B,D)**, upregulated the immunoreactivity of LC3 **(B)** and LAMP1 **(D)**, and the co-localization between mitochondria and LC3 in the axons of the RGCs **(B)**. It also accumulated co-localization between LAMP1 and mitochondria in the RGCs under glutamate excitotoxicity **(D)**. Immunofluorescence shows parkin expression in RGCs transfected with Ad null and Ad-OPA1 **(E,F)**. Compared with the Ad null transfected RGCs **(E)**, immunoreactivity of parkin were upregulated in the axons of the RGCs transfected with Ad-OPA1 **(F)**. Scale bar = 10 μm **(A–D)**. Scale bar = 25 μm **(E,F)**.

A scale of mitochondrial health was measured primarily according to the cristae appearance, and the healthiest appearance corresponds to scale of 4 (Dai et al., 2018). In comparison with the Ad null transfected RGCs, ultrastructural studies showed that Ad-OPA1-transfected RGCs showed higher mitochondrial health scores in 100 μM glutamate treatment (*P* < 0.05, **Figures 3D–F**). We next assessed mitochondrial fusion by measuring mitochondrial surface areas and nearest neighbor distance (NND). NND means the average distance between two adjacent mitochondria. It can indicate the scene of fusion or fission events. When compared to Ad null transfected RGCs, the NND was decreased in Ad-OPA1-transfected RGCs (*P* < 0.05, **Figures 3D,E,G**), while mitochondrial surface areas were increased (*P* < 0.05, **Figures 3D,E,H**). Moreover, MitoTracker Red staining showed that overexpression of OPA1 induced more number of large mitochondria around the nucleus, and partially resumed a tubular mitochondrial network (**Figures 4A,B**). The above results implied that overexpression of OPA1 reduced glutamate-induced mitochondrial fragmentation.

In addition, quantitative analysis of images from transmission electron microscopy showed the number of mitophagosomes was significantly increased in Ad-OPA1-transfected RGCs under glutamate excitotoxicity (*P* < 0.05, **Figures 3D,E,I**). Western blot analysis showed that compared with Ad null transfected RGCs, the LC3-II/I ratio were higher in Ad-OPA1-transfected RGCs under glutamate treatment (*P* < 0.05, **Figures 2C,H**). The protein level of LAMP1 was also increased in Ad-OPA1-transfected RGCs (*P* < 0.05, **Figures 2C,I**). It is noteworthy that overexpression of OPA1 upregulated the LC3 immunoreactivity and the colocalization of LC3 with mitochondria in the axons of the RGCs under glutamate excitotoxicity (**Figures 4A,B**). Moreover, overexpression of OPA1 accumulated greater immunoreactivity of LAMP1 and the colocalization of LAMP1 with mitochondria in the RGCs under glutamate excitotoxicity (**Figures 4C,D**).

### Changes in RGC Survival and Protein Expression in Chronic Hypertensive Glaucoma Rats

**FIGURE 5:**
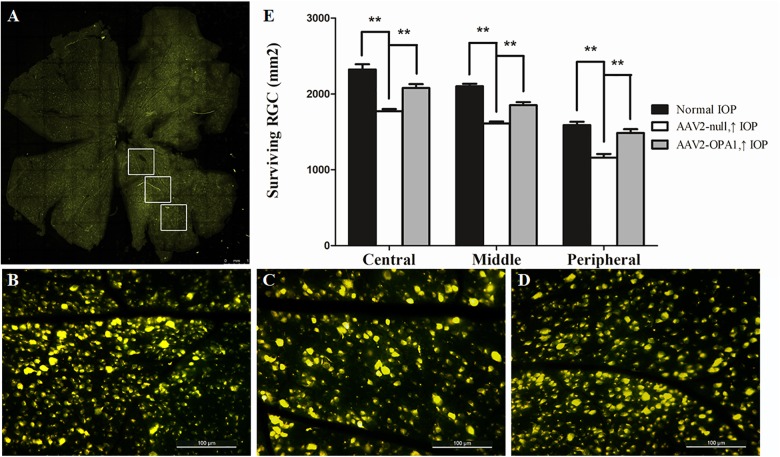
FluoroGold labeling of surviving RGCs in the hypertensive rat retina after overexpression of OPA1. Flat-mounted retinas of AAV2-OPA1-transfected hypertensive rat **(A,D)**, control rat **(B)**, and AAV2-null-transfected hypertensive rat **(C)**. Quantification of RGC survival **(E)**. *n* = 6, data are expressed as mean ± SD, ^∗∗^*P* < 0.01. Scale bar = 1 mm **(A)**, Scale bar = 100 μm **(B–D)**.

Laser treatment induced significant elevation in IOP (**Table 1**). When compared to the contralateral control eyes (**Figures 5A,B**), laser-treated eyes had a reduction in RGC loss of 28, 22, and 27% in the central, middle and peripheral retina (**Figure 5C**) 2 weeks after IOP elevation, respectively (*n* = 6 retinas, *P* < 0.01; **Figure 5E**).

**Table 1:** Intraocular pressure (IOP) exposure in the experimental glaucoma and control eyes.

Laser-treated Time(*n*)	Mean IOP (mm Hg)		Mean IOP (mm Hg)	
		
	Glaucomatous	Control	AAV2- OPA1	AAV2- null
1 Day	32.8 ± 8.7^∗∗^(*n* = 24)	10.7 ± 1.3 (*n* = 24)	30.5 ± 11.8^∗∗^ (*n* = 24)	30.0 ± 10.5^∗∗^ (*n* = 24)
3 Days	22.6 ± 5.4^∗∗^ (*n* = 24)	9.9 ± 1.0 (*n* = 24)	25 ± 9.3^∗∗^ (*n* = 24)	23.2 ± 6.8^∗∗^ (*n* = 24)
1 Week	19.4 ± 4.6^∗∗^ (*n* = 18)	10.4 ± 1.1 (*n* = 18)	20.4 ± 8.0^∗∗^ (*n* = 18)	18.6 ± 6.5^∗∗^ (*n* = 18)
2 Weeks	18.9 ± 2.8^∗∗^ (*n* = 12)	11.1 ± 0.9 (*n* = 12)	18.9 ± 6.6^∗∗^ (*n* = 12)	18.8 ± 5.6^∗∗^ (*n* = 12)


*Mean IOP is the average IOP after induction of experimental glaucoma. ^∗∗^P < 0.01 compared with control eyes.*

**FIGURE 6:**
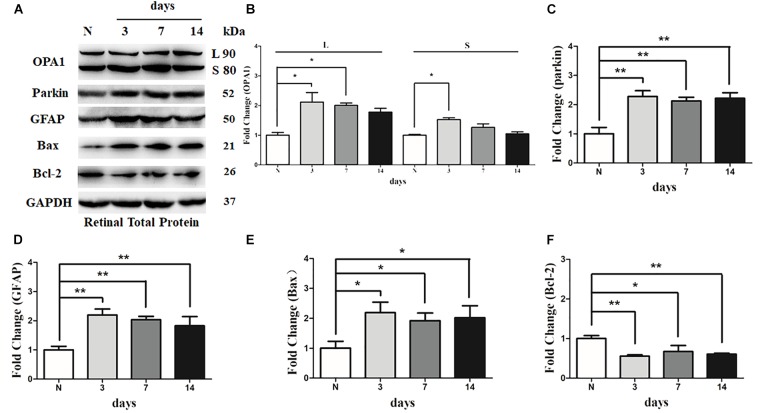
Western blot analysis of OPA1, parkin, GFAP, Bax, and Bcl-2 in hypertensive retina. Compared with control retina, expression of isoforms L and S of the OPA1 protein were increased at 3 days, and expression of isoforms L of the OPA1 protein was increased at 1 week in the hypertensive retinas **(A,B)**. Increased expression of the parkin protein **(A,C)**, GFAP protein **(A,D),** and Bax protein **(A,E)**, while decreased Bcl-2 protein **(A,F)** were observed at 3 days, 1 week, and 2 weeks in hypertensive retinas. *n* = 3, data are expressed as mean ± SD, ^∗^*P* < 0.05, ^∗∗^*P* < 0.01 compared with control retina.

There was an increase in the immunoreactivity of glial fibrillary acidic protein (GFAP) in the Muller cells in hypertensive retinas in comparison with contralateral control eyes. The results of western blot indicated that the expression level of GFAP was obviously elevated in 3 days, 1 week, and 2 weeks in hypertensive retinas (*P* < 0.01; **Figures 6A,D**).

Western blot analysis showed that the levels of long (L) and short (S) isoforms of OPA1 protein were significantly upregulated at 3 days in the hypertensive retinas after IOP elevation. In addition, the level of isoforms L of the OPA1 protein was increased 1 week in the hypertensive retinas (*P* < 0.05, **Figures 6A,B**). Compared with the control group, the protein level of Bcl2 was lower at 3 days, 1 week, and 2 weeks in hypertensive retinas (*P* < 0.05; **Figures 6A,F**), while protein levels of Bax and parkin were higher at 3 days, 1 week, and 2 weeks (*P* < 0.05; **Figures 6A,C,E**) in hypertensive retinas.

### Impacts of Increased OPA1 Expression on RGC Survival and Mitochondrial Morphology

Laser treatment induced dramatically elevated IOP in rats (**Table 1**). No statistically significant differences were detected in mean or peak IOP between two groups of AAV2-OPA1 and AAV2 null injection.

**FIGURE 7:**
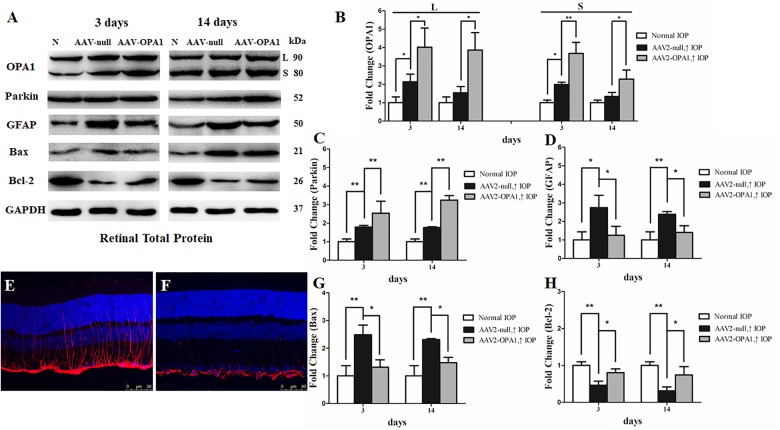
Effect of OPA1 overexpression on hypertensive retina. Western blot analysis of OPA1, parkin, GFAP, Bax, and Bcl-2 proteins in OPA1-transfected retina **(A–D,G,H)**. Compared with AAV2 null transfected retinas, expression of both the L and S isoforms of the OPA1 protein were increased in AAV2-OPA1-transfected groups at 3 days and 2 weeks **(A,B)**. Expressions of parkin **(A,C)** and Bcl-2 proteins **(A,H)** were increased, while expressions of GFAP **(A,D)** and Bax **(A,G)** proteins were decreased in AAV2-OPA1-transfected hypertensive retina at 3 days and 2 weeks. Immunofluorescence of the GFAP protein in SD rat retina **(E,F)**. Compared with AAV2 null transfected groups **(E)**, GFAP immunoreactivity was decreased in AAV2-OPA1-transfected hypertensive retinas **(F)**. *n* = 3, data are expressed as mean ± SD, ^∗^*P* < 0.05, ^∗∗^*P* < 0.01. Scale bar = 50 μm **(E,F)**.

To assess the efficiency of transgene expression, immunoblotting was used to detect the levels of OPA1 in retinas. Compared with AAV2 null transfected groups, both two isoforms of the OPA1 protein expression was greatly elevated in AAV2-OPA1-transfected groups at 3 days, 1 week, and 2 weeks in the hypertensive retinas (*P* < 0.05, **Figures 7A,B** and **Supplementary Figures S4A,B**).

Compared with AAV2 null transfected hypertensive rats, overexpression of OPA1 had an obvious positive effect on RGC survival: nearly increased 18, 15, and 28% viable RGCs in the central, middle, and peripheral retina, respectively (*n* = 6 retinas, *P* < 0.01; **Figures 5D,E** and **Supplementary Figure S3**).

The results of western blot and immunofluorescence indicated that compared with AAV2 null transfected groups, the expression of GFAP protein was lower in AAV2-OPA1-transfected hypertensive retinas at 3 days, 1 week, and 2 weeks (*P* < 0.05; **Figures 7A,D–F** and **Supplementary Figures S4A,C**). The expression of Bax protein was lower in AAV2-OPA1-transfected hypertensive retinas at 3 days, 1 week, and 2 weeks (*P* < 0.05; **Figures 7A,G**) while the expression of Bcl-2 protein was higher in AAV2-OPA1-transfected hypertensive retina at 3 days and 2 weeks (*P* < 0.05; **Figures 7A,H**).

**FIGURE 8:**
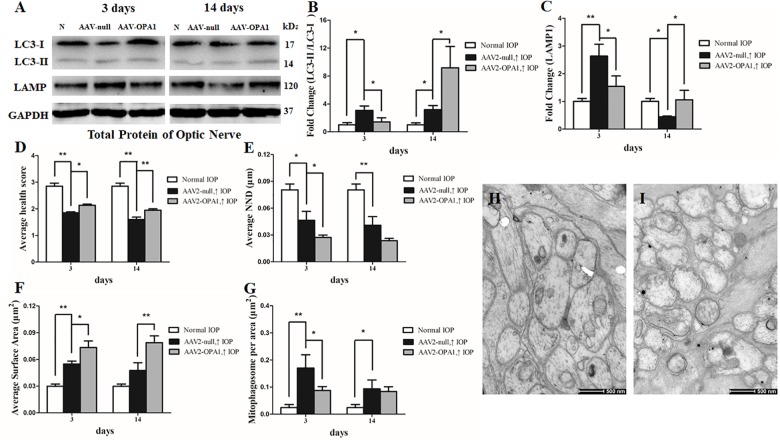
Impacts of OPA1 overexpression on mitophagy in hypertensive optic nerve. Western blot analysis of LC3-I and -II and LAMP1 proteins in OPA1-transfected optic nerve **(A–C)**. Compared with AAV2 null transfected groups, LC3-II/I ratio **(A,B)**, and the expression of LAMP1 protein **(A,C)** in AAV2-OPA1-transfected hypertensive optic nerves were decreased at 3 days, yet increased at 2 weeks. Ultrastructural analysis of mitochondrial morphology and mitophagy in hypertensive optic nerves **(D–I)**. Compared with the AAV2 null transfected groups **(H)**, higher mitochondrial health scores **(D,I)**, closer nearest neighbor distance (NND) **(E,I)**, and larger mitochondrial surface areas **(F,I)** were observed at 3 days and 2 weeks in AAV2-OPA1-transfected groups. The number of mitophagosomes was decreased at 3 days **(G,I)**. *n* = 3, data are expressed as mean ± SD, ^∗^*P* < 0.05, ^∗∗^*P* < 0.01. Scale bar = 500 nm **(H,I)**.

As shown in transmission electron microscopy analysis in **Figure 8**, higher mitochondrial health scores were observed in AAV2-OPA1 -transfected groups (*P* < 0.05; **Figures 8D–I**). In comparison with AAV2 null transfected groups, the NND was reduced in AAV2-OPA1-transfected groups at 3 days (*P* < 0.05; **Figures 8E,H,I**), no statistically significant change (*p* > 0.05) was observed in NND at 2 weeks. While mitochondrial surface areas were larger at 3 days and 2 weeks (*P* < 0.05; **Figures 8F,H,I** and **Supplementary Figures S4D–F**). The increases in mitochondrial surface area amount to 33.8 and 65.6% larger surface areas at 3 days and 2 weeks than the corresponding AAV2 null transfected groups. Dramatically reduction in the number of mitophagosomes was observed at 3 days (*P* < 0.05; **Figures 8G–I**), yet no significant change was observed at 2 weeks.

Moreover, in comparison with AAV2 null transfected groups, LC3-II/I ratio was decreased at 3 days in AAV2-OPA1-transfected hypertensive optic nerve, yet was increased at 2 weeks following IOP elevation (*P* < 0.05; **Figures 8A,B**). The expression of LAMP1 protein was downregulated at 3 days in AAV2-OPA1-transfected hypertensive optic nerves, but upregulated at 2 weeks following IOP elevation (*P* < 0.05; **Figures 8A,C**).

### Overexpression of OPA1 Upregulated the Expression of Parkin Under Glutamate Excitotoxicity and in Chronic Hypertensive Glaucoma Rats

Compared with Ad null transfected RGCs, western blot analysis showed that Ad-OPA1-transfected RGCs increased the protein level of parkin in neurobasal medium and in glutamate treatment (*P* < 0.05, **Figures 2C,G**). It also upregulated the immunoreactivity of parkin in the axons of the RGCs (**Figures 4E,F**).

To further investigate whether overexpression of OPA1 alter the expression level of parkin, we analyzed the western blot results in hypertensive retina of rats. In comparison with the AAV2 null transfected groups, the expression of parkin protein was increased in AAV2-OPA1-transfected hypertensive retinas at 3 days, 1 week, and 2 weeks (*P* < 0.01; **Figures 7A,C**).

### Knockdown of OPA1 Down-Regulated the Expression of Parkin and Related Proteins Under Glutamate Excitotoxicity

Cultured RGCs were transfected with OPA1 siRNA to knockdown OPA1 gene expression. Both mRNA and protein levels of OPA1 were decreased in the RGCs transfected with OPA1 siRNA (*P* < 0.05; **Supplementary Figures S1D, S2A,B**.

To investigate the effect of OPA1 knockdown on RGC viability, Hoechst-staining, LDH assay, JC-1 assay and western blotting were performed. Hoechst staining revealed that the knockdown of OPA1 caused increases in apoptosis of RGCs compared the effect observed in the siRNA-control group with 0 μM or 100 μM glutamate treatment (*P* < 0.05; **Supplementary Figure S1A**). LDH measurements showed similar results and indicated that knockdown of OPA1 increased cytotoxicity in RGCs with 0 μM or 100 μM glutamate treatment (*P* < 0.05; **Supplementary Figure S1B**). The JC-1 ratio was statistically significantly decreased in siRNA-OPA1 group with 0 μM or 100 μM glutamate treatment (*P* < 0.05; **Supplementary Figure S1C**). Besides, western blot analysis showed that the knockdown of OPA1 increased the level of Bax protein, while decreased the level of Bcl-2 protein (*P* < 0.05; **Supplementary Figures S2A,C,D**). These results suggested that knockdown of OPA1 leads to the apoptosis and cytotoxicity in RGCs.

Compared with siRNA-control group, knockdown of OPA1 decreased mRNA expression of PINK, parkin, LC3, and LAMP1 under 100 μM glutamate treatment (*P* < 0.05; **Supplementary Figure S1D**). It also reduced the protein levels of PINK, parkin, LAMP1 and LC3-II/I ratio (*P* < 0.05; **Supplementary Figures S2A,E–H**) under 100 μM glutamate treatment. These data indicated that knockdown of Opa1 leads to the downregulation of parkin mediated mitophagy pathway.

## Discussion

These results demonstrated that overexpression of OPA1 protected against RGC loss in chronic hypertensive rats *in vivo* and in glutamate induced excitotoxicity *in vitro*. While knockdown of OPA1 decreased RGC viability and parkin expression in RGCs. Increased OPA1 expression attenuated Bax expression, improved mitochondrial health and mitochondrial surface area in experimental glaucoma. Parkin expression and parkin mediated mitophagy were upregulated in OPA1 overexpressed optic nerve in response to cumulative IOP elevation.

Mitochondrial fusion restores dysfunctional mitochondria by merging functional mitochondria, and it can mitigate toxic stress on cell. Fusion between mitochondrial inner membranes is mediated by OPA1 (Youle and van der Bliek, 2012). Given its role in mitochondrial fusion, one could predict that OPA1 reduces mitochondrial dysfunction, cytochrome c release, and cell death by counteracting apoptotic fragmentation of mitochondria (Youle and Karbowski, 2005; Jahani-Asl et al., 2011). Previously, it has been shown that increased expression of OPA1 promoted RGC survival in a mouse model of glaucoma, and prevented mitochondrial fission and promoted RGC-5 survival following elevated hydrostatic pressure as well (Ju et al., 2010). Since recent evidences demonstrated that RGC-5 cells were not of RGC origin, but similar to 661W cells of cone photoreceptor origin from mouse (Krishnamoorthy et al., 2013). Any data derived from RGC-5 cells must be carefully interpreted. In this study, purified RGCs from rats were used to investigate the role of OPA1 in regulating mitochondrial homeostasis of RGCs under glutamate excitotoxicity. We found that overexpression of OPA1 significantly decreased cytotoxicity, apoptosis and Bax expression, increased the mitochondrial membrane potential, mitochondrial health and mitochondrial surface area in RGCs under excitotoxicity. While knockdown of OPA1 increased cytotoxicity, apoptosis and Bcl-2 expression and decreased the mitochondrial membrane potential in RGCs. In addition, laser photocoagulation induced chronic hypertensive glaucoma rats were used in this study, the level and duration of IOP elevation differed from that of DBA/2J glaucomatous mice. We found that overexpression of OPA1 increase RGC survival in chronic hypertensive rats. With regard to the mitochondrial morphology, a significant upregulation of mitochondrial health and mitochondrial surface area, yet a down-regulation of NND at 3 days were observed in optic nerve. Taken together, our data are consistent with the notion that OPA1 exerted a significant protective effect on RGCs via promoting mitochondrial fusion in experimental glaucoma.

Bax is a pro-apoptotic member of the Bcl-2 family, which is critical in the mitochondrial apoptotic pathway. Bax translocates from cytosol to the mitochondria and controls mitochondrial outer membrane permeabilization (MOMP) (Grosse et al., 2016) which permit proteins, including cytochrome C, to release from the mitochondrial intermembrane space into the cytosol to initiate apoptosis (Desagher and Martinou, 2000). Conversely, antiapoptotic Bcl-2, another essential protein of the Bcl-2 family, inhibit the release of cytochrome C, and ultimately contribute to inhibits apoptosis (Brunelle and Letai, 2009). We provided both *in vivo* and *in vitro* evidences that overexpression of OPA1 attenuated the Bax expression and partially increased the Bcl-2 expression. Interestingly, a recent study reported that an N-terminal fragment of OPA1 can inhibit MOMP and decrease Bax activation (Kushnareva et al., 2016). Previous studies have suggested that direct interaction between Bcl-2 family members and Mfn1/2 may promote mitochondrial fusion (Martinou and Youle, 2011). These data support the assumption that the antiapoptic effect of OPA1 on RGCs may be mediated by regulating the function of Bax and Bcl-2. Further studies will be needed to clarify the molecular mechanism among OPA1, Bax, and Bcl-2 in experimental glaucoma.

Recently, it has been suggested that mitophagy is functionally associated with mitochondrial dynamics via the interaction between mitochondrial dynamic proteins and LC3 adapter/receptors (Yoo and Jung, 2018). OPA1 may also play a role as a mitophagic factor (Yoo and Jung, 2018). On the other hand, growing evidences indicate that mitophagy exerts a protective effect via selective removal of damaged mitochondria in a variety of pathological models. When the mitochondrial membrane potential is depolarized by irreversible damage, parkin is recruited to the outer mitochondrial membrane, which recruits the phagophore protein LC3, inducing autophagosome formation around the organelle (Vives-Bauza et al., 2010). LC3-II/I ratio has been used to evaluate the induction of autophagy (Coughlin et al., 2015). Increased parkin and LAMP1 level and LC3-II/I ratio indicate enhanced cycling of parkin-mediated mitophagy. Our previous results suggested that mitophagy may be critical for the neuroprotective effect of parkin in experimental glaucoma (Dai et al., 2018). Since the mechanism of mitochondrial fusion regulated by OPA1 could not fully explain the neuroprotective effect of OPA1, and the link between mitochondria fusion and mitophagy is not fully understood, we investigated the role of parkin-mediated mitophagy in OPA1 overexpressed experimental glaucoma models. Frezza et al. (2006) reported that OPA1 regulated mitochondrial cristae remodeling and protected from apoptosis, and this occurs independently from mitochondrial fusion. The current study found that parkin expression, the LC3-II/I ratio and the number of mitophagosomes were upregulated in OPA1 overexpressed RGCs under glutamate excitotoxicity. While LC3-II/I ratio and the LAMP1 expression were decreased in OPA1 overexpressed optic nerve at 3 days, then increased at 2 weeks after IOP elevation. Our data further showed that knockdown of OPA1 by siRNA decreased protein expression of parkin, as well as LC3-II/I ratio and LAMP1, in RGCs under glutamate excitotoxicity. Together with these findings, our results suggest that the mechanism by which OPA1 mediated RGC protection in experimental glaucoma may not only through enhancing mitochondrial fusion, but also relying on the parkin-mediated mitophagy. Further studies are needed to clarify the molecular pathway involving OPA1 and parkin in glaucomatous RGCs.

In summary, our findings demonstrate that overexpression of OPA1 protects RGCs by ways of enhancing mitochondria fusion and parkin mediated mitophagy in experimental glaucoma. Interventions to promote mitochondrial fusion and mitophagy may provide a useful strategy to battle against glaucomatous RGC loss.

## Author Contributions

XS and YD designed the experiments and revised the manuscript. XH and YD did the experiments and drafted the manuscript. RZ and KS took part in some of the experimental studies, for example, immunohistochemistry and confocal microscopy. All authors read and approved the final manuscript.

## Conflict of Interest Statement

The authors declare that the research was conducted in the absence of any commercial or financial relationships that could be construed as a potential conflict of interest.
