# Supplementary figures and images for: Overexpression of Optic Atrophy Type 1 Protects Retinal Ganglion Cells and Upregulates Parkin Expression in Experimental Glaucoma
Source: Front Mol Neurosci. 2018 Sep 28;11:350. doi: 10.3389/fnmol.2018.00350 (PMC6172338; doi:10.3389/fnmol.2018.00350)

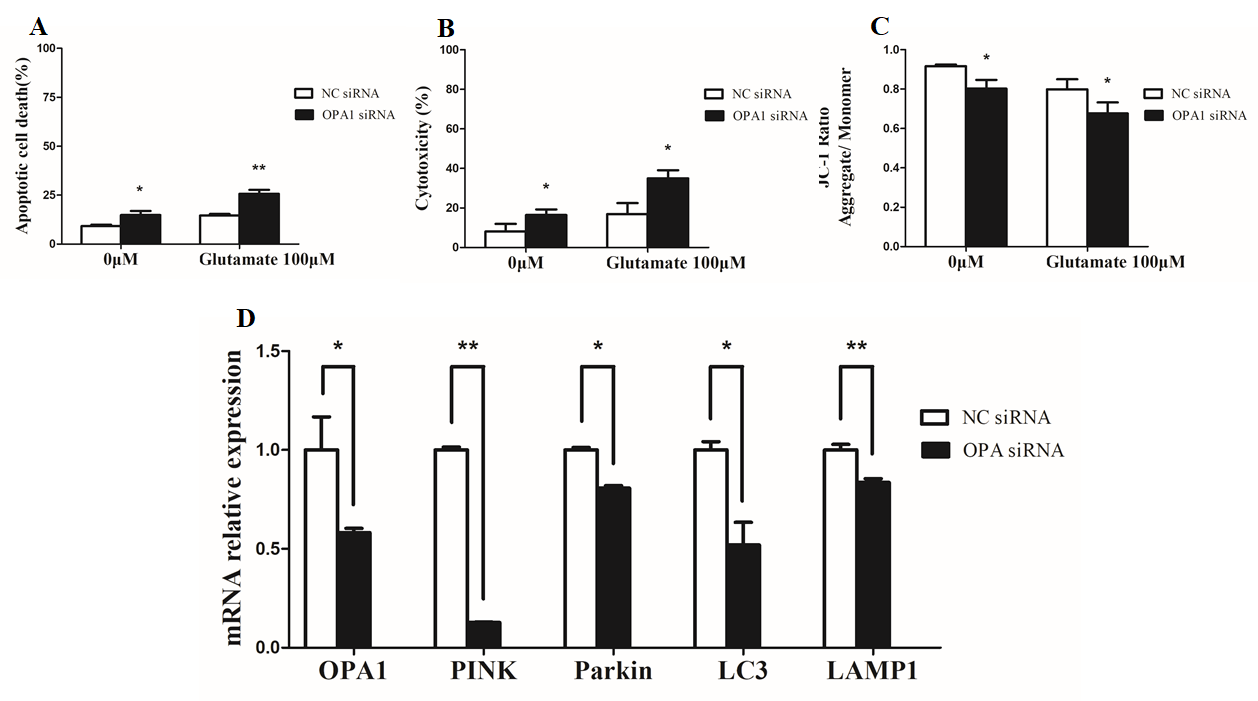

Supplement: Supplementary file 1 [file Image_1.TIF]

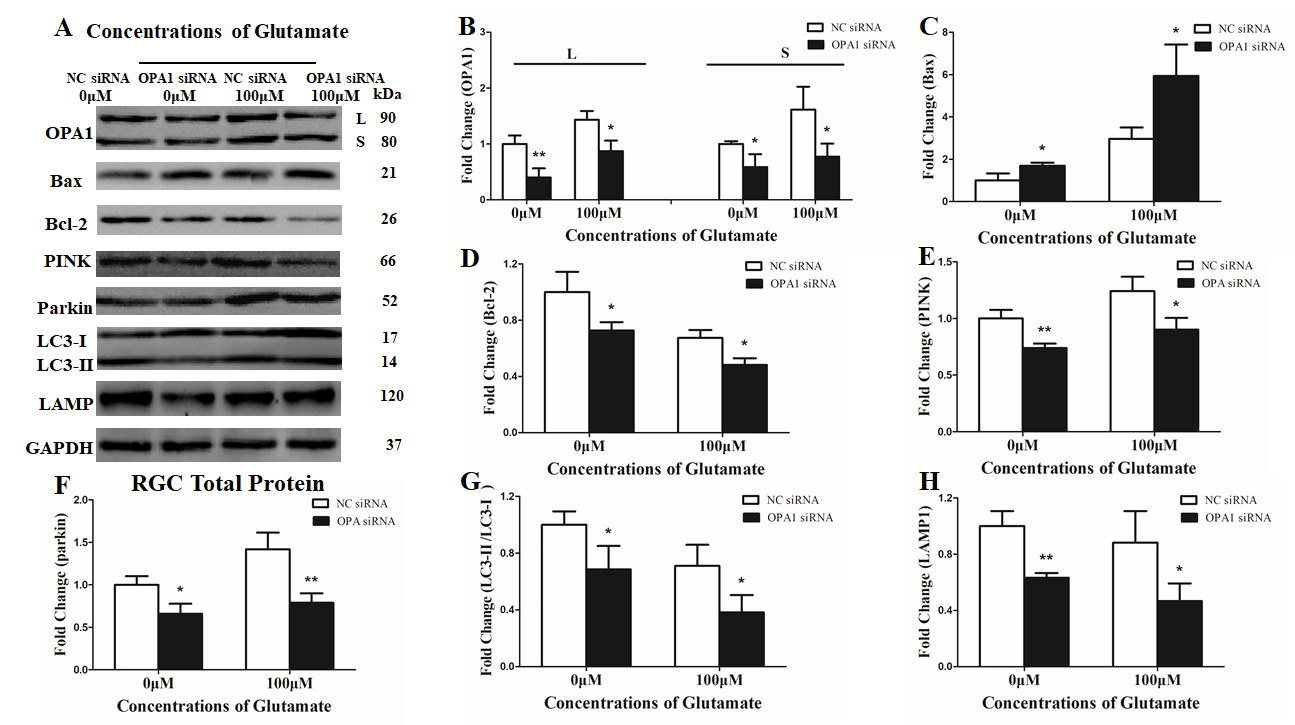

Supplement: Supplementary file 2 [file Image_2.TIF]

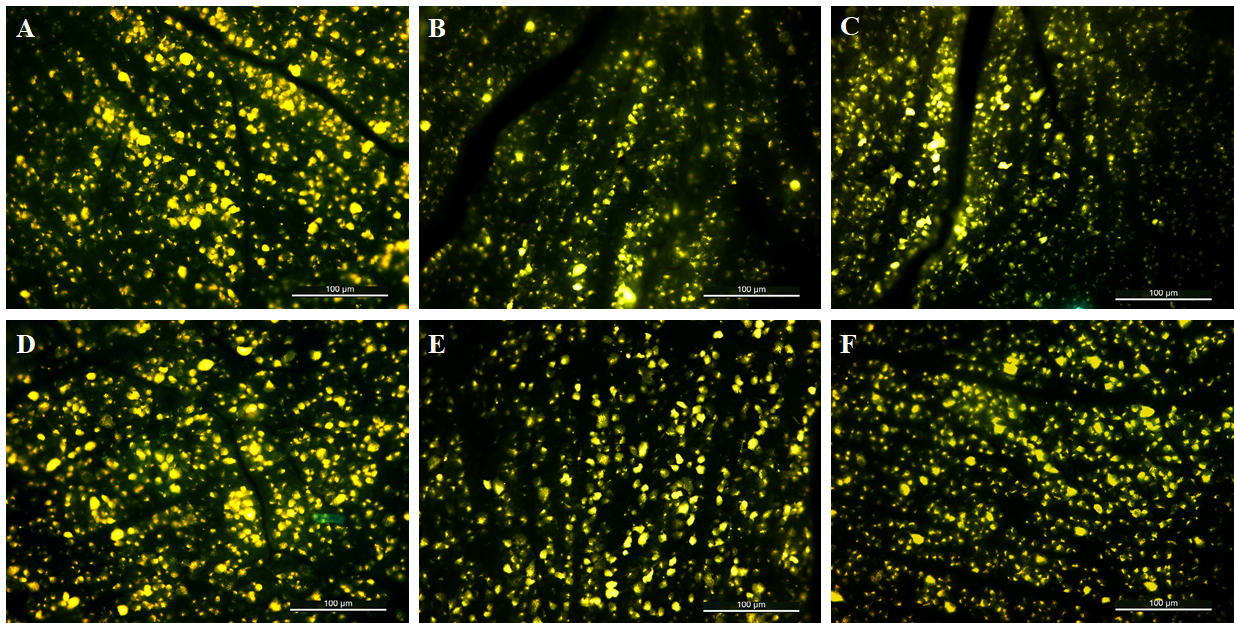

Supplement: Supplementary file 3 [file Image_3.TIF]

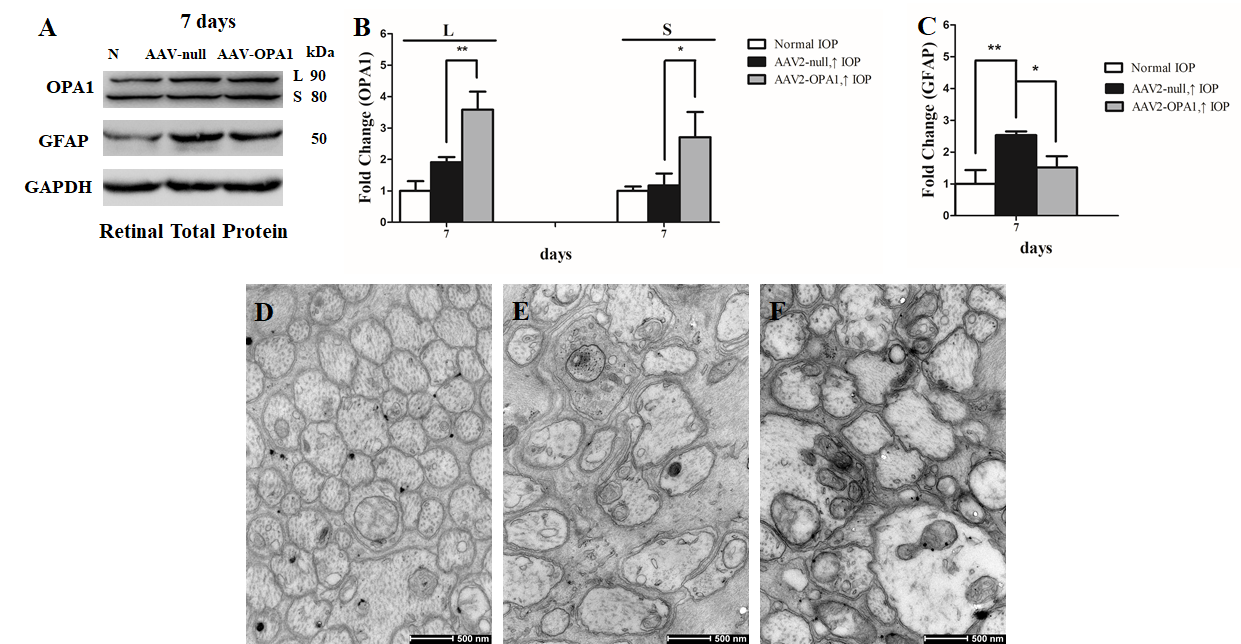

Supplement: Supplementary file 4 [file Image_4.TIF]
